# ANTIBIOTIC EXPOSURE IN LONG-TERM CARE FACILITIES IS ASSOCIATED WITH URINARY MICROBIOME PERTURBATIONS

**DOI:** 10.1093/geroni/igz038.331

**Published:** 2019-11-08

**Authors:** Brent R Schell, Doyle V Ward, Evan S Bradley, Protiva Dutta, John P Haran

**Affiliations:** University of Massachusetts Medical School, Worcester, Massachusetts, United States

## Abstract

The discovery of the human urinary microbiome, defined as the microbial communities which colonize the human urinary tract, has shed new light on the meaning and clinical significance of bacteriuria. The prevalence of asymptomatic bacteriuria has been reported to be as high as 50% in healthy older adults living in long term care facilities, yet the urinary microbiome of this population has not been reported. The aim of this pilot study was to describe the urinary microbiome of this population and explore the cross-sectional relationship with recent antibiotic exposure. Voided urine samples were obtained from healthy, institutionalized older adults (ages 79 to 95), including non-catheterized men and women without any urinary symptoms. The bacterial genomic content of each urine sample was assessed by 16S rRNA sequencing. Among the 77 genera found across 16 urine samples, there was no significant difference in the microbial diversity across age groups. When grouped by antibiotic exposure, those recently exposed had a significantly lower diversity (Shannon’s index of 2.16 vs. 2.61, p = 0.029), and lower evenness (Pielou’s evenness of 0.58 vs. 0.69, p= 0.017) relative to those who were not recently exposed. Enrichment analysis showed that recent antibiotic exposure was associated with an increased abundance of the genus Bacteroides and decreased abundance of the genus Streptococcus. To our knowledge, this is the first report describing the urinary microbiome of institutionalized older adults and suggests that recent antibiotic exposure should be accounted for in future studies of the urinary microbiome in this population.

